# Exploring the interplay of trunk and shoulder rotation strength: a cross-sport analysis

**DOI:** 10.3389/fphys.2024.1371134

**Published:** 2024-04-26

**Authors:** Mikulas Hank, Petr Miratsky, Kevin R. Ford, Christian Clarup, Osman Imal, Ferdia Fallon Verbruggen, Frantisek Zahalka, Tomas Maly

**Affiliations:** ^1^ Sport Research Center, Faculty of Physical Education and Sport, Charles University, Prague, Czechia; ^2^ Department of Physical Therapy, High Point University, High Point, NC, United States; ^3^ Performance Department, AC Sparta Praha, Prague, Czechia

**Keywords:** isometrics, muscular strength, performance, optimization, injury prevention, adaptation, professional athletes

## Abstract

**Introduction:** Trunk and shoulder strength are consistently shown to be involved in performance limitations, as well as contributing to stability, power output, and reducing the risk of injury. Although their biomechanical interaction is a critical aspect for athletes, there is limited research on the relationship between trunk and shoulder strength in sports where upper body mechanics are critical for optimal performance.

**Purpose:** This study examined the differences and relationships between trunk rotational strength and shoulder rotational strength among athletes participating in mixed martial arts (MMA), tennis, swimming, and baseball.

**Methods:** Maximal voluntary contraction tests were performed to evaluate strength of 39 professional adult male athletes from disciplines of MMA (*n* = 6), tennis (*n* = 11), swimming (*n* = 11) and baseball (*n* = 11). Peak force data were used in sports comparison and relationship analysis between trunk and shoulder rotation strength parameters.

**Results:** The findings revealed a complex and significant relationship between trunk and shoulder strength, with unique patterns for each athletic discipline. Tennis players exhibited a strong correlation between trunk bilateral differences and internal shoulder rotation, while other disciplines demonstrated a more balanced use of trunk asymmetry. Swimmers displayed the best interactions between trunk and shoulder overall, emphasizing the aquatic environment’s biomechanical demands. In MMA, the strongest correlation was between shoulder internal and external rotation with the trunk, mainly due to the number of defensive movements in addition to offensive ones. Baseball pitchers showed a significant correlation between internal/external shoulder rotation strength ratio and trunk asymmetry.

**Conclusion:** While no differences in peak force variables were found, unique relationships between trunk and shoulder rotational performance were discovered. The results suggest a long-term sport-specific adaptation of the trunk-shoulder interaction in sports that require upper limb power movements. It seems, that the relationship between the various parameters of trunk and shoulder was influenced by the movement stereotype of each sport. Therefore, recognition of sport-specific interactions is critical to the development of effective training programs that enhance performance and potentially reduce injury risk in different sports. Researchers and practitioners should focus on longitudinally monitoring fluctuations in TRS and SRS relationships throughout each sport season and examining potential associations with injury incidence.

## 1 Introduction

The interaction between trunk rotational strength (TRS) and shoulder rotational strength (SRS) is a critical aspect of athletic performance in various sports, where pelvis and upper body mechanics are critical for optimal performance during locomotion or ballistic throwing (Eckenrode et al., 2012; Weber et al., 2014; Zenovia et al., 2016; Kaurkin et al., 2020; Sioutis et al., 2022). The biomechanical underpinnings of athletic movements also have implications for training strategies (Prieske et al., 2016), injury prevention (Peate et al., 2007; Wright et al., 2021), and performance optimisation (Hibbs et al., 2008) in sports. The trunk acts as a kinetic chain linking the lower and upper extremities, facilitating power transmission and rotational power during complex movements such as mixed martial arts (MMA), tennis, swimming, or baseball (Liebenson, 2010; Çetinkaya, 2015; Zemková et al., 2020; Mornieux et al., 2021). Additionally, trunk strength was correlated with performance limitations and contributes to stability, high performance, and reduced injury risk (Roth, 2019). In tennis, the ability to execute powerful serves and rapid rotational movements directly correlates with TRS (Baiget et al., 2016). Similarly, in MMA, effective striking and grappling manoeuvres rely on the athlete’s ability to generate force through rapid trunk rotations (Tong-lam et al., 2017; Dunn et al., 2022). Swimmers, who participate in a sport predominantly characterised by upper body movements, require robust trunk rotation to optimise their strokes and maintain streamlined body positions (Lawsirirat and Chaisumrej, 2017). Baseball athletes, specifically during pitching, rely on the synchronisation of trunk and shoulder rotations to unlock the full potential of throwing motions (Bullock et al., 2018). Meanwhile, the role of SRS in these sports should not be overlooked. Research continues to highlight the role of SRS in improving performance and preventing overuse and imbalance related injuries (Payton et al., 2002; Liebenson, 2010; Lawsirirat and Chaisumrej, 2017). The shoulder complex is intimately involved in various sport-specific movements, such as the execution of powerful strikes in MMA (Zhou et al., 2023), the rapid rotational movements of overhead serve in tennis (Gordon and Dapena, 2006), the propulsive arm strokes in swimming (Collado-Mateo et al., 2018), and the high-speed throws in baseball (Cross et al., 2023). Despite the apparent commonality in the need for TRS, the specific demands of each sport may result in unique musculoskeletal stressors and adaptations (Stefan et al., 2015), requiring a harmonious interplay between the rotational forces generated by the trunk and shoulders (Zenovia et al., 2016; Kaurkin et al., 2020; Sioutis et al., 2022). Although individual studies have focused on either TRS or SRS measurements in specific sports, there is a research gap in comprehensively examining their interrelationship across multiple sport disciplines. Therefore, this study investigated the trunk and shoulder rotational strength differences and relationships between trunk and shoulder variables in athletes from various sports, including MMA, tennis, swimming, and baseball. The goal was to gain a better understanding of how these strengths interacted and influenced athletic performance. We hypothesized significantly different strength performance of trunk and shoulder between sports. Additionally, we expected significant correlations between trunk and shoulder strength variables. A deeper explanation of the relationships between these factors can provide valuable and novel insights in the fields of sports science and performance enhancement. Additionally, the development of new tailored training regimes can help athletes optimize their performance and reduce the risk of overuse injuries and musculoskeletal imbalances associated with the repetitive rotational movements and high-impact nature of their sports.

## 2 Materials and methods

### 2.1 Study design

This was a cross-sectional study. All participants were fully informed of the research procedures and agreed to the experimental design by signing an informed consent form. This research was approved by the ethic committee of Faculty of Physical Education and Sport at Charles University, and was in accordance with the Declaration of Helsinki guidance.

### 2.2 Participants

A total of 39 adult male professional athletes were included: 6 MMA athletes at the highest national competitive level, 11 tennis players at the international and national competitive level, 11 swimmers at the highest international and national competitive level, and 11 baseball pitchers at the Czech Republic national team level. Descriptive group characteristics are presented in Table 1. Dominant upper limb categorisation was based on verbal questioning. To be included in this study, athletes had to be free from any musculoskeletal injuries or medical conditions that would exclude them from competition and training; no high resistance and exhausting physical activity in the last 48 h prior testing.

**TABLE 1:** Descriptive research group characteristics.

		Mean	Std. Deviation	Std. Error	95% CI for mean	
					Lower bound	Upper bound
Age (years)	Baseball	28.27^a, b^	5.55	1.67	24.54	32.00
	MMA	26.83	8.54	3.49	17.87	35.80
	Swimming	21.46^a^	2.54	0.77	19.75	23.16
	Tennis	22.18^b^	3.60	1.09	19.76	24.60
BH (cm)	Baseball	184.45	6.89	2.08	179.83	189.08
	MMA	183.17	4.36	1.78	178.60	187.74
	Swimming	185.03	4.00	1.21	182.34	187.71
	Tennis	185.55	6.86	2.07	180.94	190.15
BW (kg)	Baseball	84.82	10.25	3.09	77.93	91.71
	MMA	80.55	7.11	2.90	73.09	88.01
	Swimming	81.25	3.67	1.11	78.79	83.72
	Tennis	81.83	7.48	2.25	76.80	86.85

Note: BH, body height; BW, body weight.

^a, b^–Bonferroni *post hoc* test (same letter means that both groups are significantly different each other.

### 2.3 Data collection

#### 2.3.1 Isometric trunk rotational strength

**FIGURE 1:**
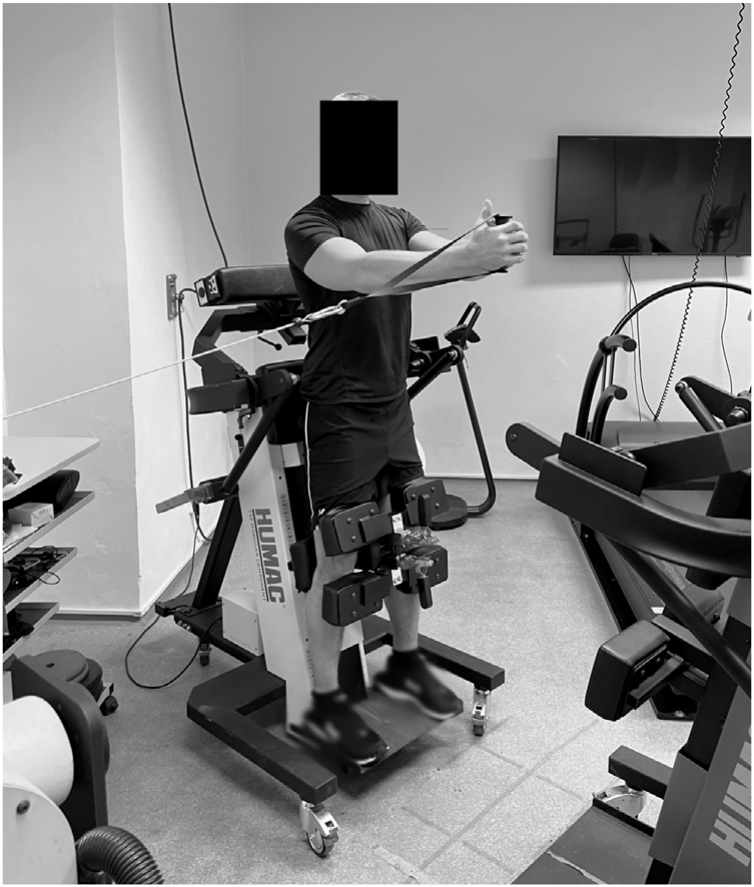
Maximal voluntary contraction test of trunk rotational strength using isometric mode of Humac NORM dynamometer with Trunk Modular and Wheel Components (CSMi, Stoughton, MA).

Maximal voluntary contraction (MVC) of the TRS was assessed using the Humac NORM dynamometer (CSMi, Stoughton, MA) in isometric mode. A standardised warm-up of 3 × 6 repetitions (each side) of Bird Dog; 3 × 5 seconds (each side) of Pallof Press with elastic band and 3 × 6 roll-up crunch was performed prior to TRS testing. The study utilized a variant of the anti-rotation pallof press (Mullane et al., 2021) in a vertical position with as many degrees of freedom as possible. This was done because the force of trunk rotation in the serape effect is closely linked to the movement of the entire body, including the lower limbs and pelvic region. To execute maximal force without shearing the feet, fixation was chosen in the knee region. Pilot measurements with fixation in the ankle region caused too much stress and discomfort when achieving sub-maximal to maximal forces. The closest possible point was at the knee area, which proved to be ideal and stable. For testing, participants stood upright in the Trunk Modular Component (CSMi, Stoughton, MA) with their knees fixed to prevent lower limb movement and allow for maximal effort trials (Figure 1). The Trunk Modular Component was adjusted to individual height until the dynamometer attachment reached the celiac plexus. Both hands held the Humac NORM Wheel (CSMi, Stoughton, MA) at shoulder height with the arms straight (no elbow flexion was allowed during the test). Starting testing side was decided by freeware online random number generator (1-right; 2-left) to prevent the order effect. Depending on testing side, the hand closer to the dynamometer was always on top. For familiarisation, participants performed 2 submaximal trials of 3 s separated by 30-s rest. The MVC protocol consisted of 4 maximal 3-s trials separated by a 60-s rest. The test was performed on the dominant and non-dominant side. Evaluated variables were absolute TRS for dominant (TRD) and non-dominant side (TRN), TRS normalized according to body weight in percentages for dominant (TRDrel) and non-dominant side (TRNrel), and TRS asymmetry in percentages (TRdiff).

#### 2.3.2 Isometric shoulder rotational strength

**FIGURE 2:**
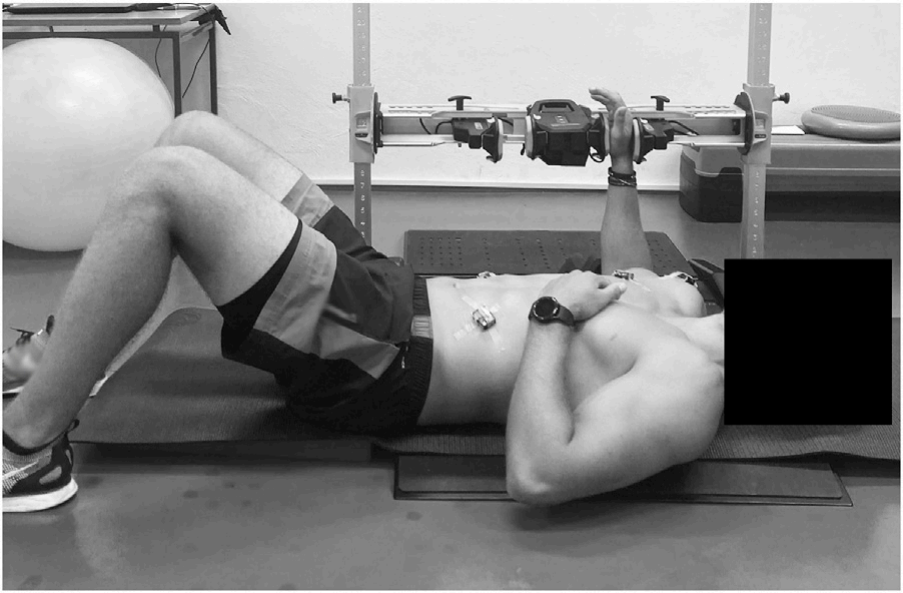
Maximal voluntary contraction test of shoulder internal and external rotational strength using isometric dynamometer ForceFrame (Vald, United States) in supine body at 90° shoulder abduction and 90° elbow flexion.

MVC of internal and external rotational SRS was assessed using the ForceFrame isometric dynamometer (Vald Performance, Albion, Australia). Besides individual shoulder mobilization before SRS testing, a standardised warm-up of 3 × 10 repetitions (each side) of internal and external shoulder rotation with a medium resistance elastic band was performed. For testing, the body was positioned in the supine position with 90° of shoulder abduction and 90° of elbow flexion, while bending the knees at 90° (Figure 2). For familiarisation, participants performed 3 submaximal trials of 3 s separated by 30 s rest. The MVC protocol consisted of 3 maximal 3-s trials of internal and external rotation in the dominant upper limb separated by a 60-s rest. Evaluated variables were absolute internal and external SRS for dominant upper limb (IRD; ERD), SRS normalized according to body weight in percentages for dominant upper limb (IRDrel; ERD_rel_), and SRS ratio between external and internal rotation (IRD:ERD).

### 2.4 Data processing

Statistical analysis was performed using IBM SPSS v24 (Statistical Package for Social Sciences, Inc., Chicago, IL, United States). Basic descriptive statistics (Mean and Std. Deviation) were calculated for all dependent variables. Normal data distribution was evaluated and confirmed using the Shapiro-Wilk test, while homogeneity of variance was tested with Levene’s test. Pairwise comparison was calculated by independent T-Test with 95% CI, while multiple comparison was calculated by One-way Between-groups ANOVA with 95% CI. Statistical significance was set at *p* < 0.05. Relationships between the dependent variables were calculated by Pearson’s correlation. Explanation of the proportion of factor variance (effect size) was evaluated by the Partial Eta Squared (*η*_*p*_^*2*^) as small 0.01, medium 0.06 and large 0.14 (Richardson, 2011).

## 3 Results

### 3.1 Isometric trunk rotational strength

Intra-class correlation coefficient in TRS (ICC = 0.979) with standard error of measurement = 3.07 (%SEM = 13.38%) and minimum detectable change = 4.86 (SDC% = 21.17%) was calculated prior to analysis. There were no statistically significant differences between groups in TRS parameters (*p* > 0.05, Table 2). Higher, but non-significant differences were found in TRdiff between MMA (9.83% ± 8.33%), tennis players (9.73% ± 7.75%) compared to swimmers (5.12% ± 3.09%) and baseball pitchers (6.36% ± 3.75%).

**TABLE 2:** Differences in isometric trunk rotational strength.

		Mean	Std. Deviation	Std. Error	95% CI for mean		F	*p*
					Lower bound	Upper bound		
TRD (kg)	Baseball	23.64	4.80	1.45	20.41	26.86	0.46	0.71
	MMA	24.17	6.59	2.69	17.26	31.08		
	Swimming	22.09	2.70	0.81	20.28	23.90		
	Tennis	22.36	3.70	1.11	19.88	24.85		
TRN (kg)	Baseball	24.73	4.96	1.50	21.39	28.06	1.44	0.25
	MMA	22.67	4.23	1.73	18.23	27.10		
	Swimming	21.55	2.25	0.68	20.03	23.06		
	Tennis	24.27	4.13	1.24	21.50	27.04		
TRdiff (%)	Baseball	6.36	3.75	1.13	3.85	8.88	1.62	0.20
	MMA	9.83	8.33	3.40	1.09	18.57		
	Swimming	5.12	3.09	0.93	3.04	7.20		
	Tennis	9.73	7.75	2.34	4.52	14.93		
TRDrel (%BW)	Baseball	28.06	4.33	1.31	25.15	30.97	0.22	0.89
	MMA	29.00	6.60	2.70	22.07	35.93		
	Swimming	27.15	2.92	0.88	25.19	29.11		
	Tennis	27.64	5.30	1.60	24.08	31.19		
TRNrel (%BW)	Baseball	28.61	3.18	0.96	26.47	30.74	1.55	0.22
	MMA	29.00	4.24	1.73	24.55	33.45		
	Swimming	26.54	2.60	0.78	24.79	28.29		
	Tennis	30.09	5.30	1.60	26.53	33.65		

Note: TRD, trunk rotational strength for dominant side; TRN, trunk rotational strength for non-dominant side; TRD_rel_, trunk rotational strength to body weight for dominant side; TRN_rel_, trunk rotational strength normalized to body weight for non-dominant side; TRdiff, trunk rotational strength asymmetry between dominant and non-dominant side.

### 3.2 Isometric shoulder rotational strength

SRS parameters were not statistically different between groups (*p* > 0.05, Table 3). While MMA athletes were able to generate similar strength in IRDrel (2.14 ± 0.43 %BW) and ERDrel rotation (2.12 ± 0.40 %BW), tennis players had up to 11.32% stronger IRDrel (2.12 ± 0.37 %BW) compare to ERDrel (1.88 ± 0.30 %BW). The highest value of IRDrel was 2.85 %BW in swimmer and lowest values (1.18 %BW) has been detected in baseball pitcher.

**TABLE 3:** Differences in isometric shoulder rotational strength.

		Mean	Std. Deviation	Std. Error	95% CI for mean		F	*p*
					Lower bound	Upper bound		
IRD (N)	Baseball	153.59	47.43	14.30	121.73	185.45	0.51	0.68
	MMA	172.79	43.14	17.61	127.52	218.06		
	Swimming	169.66	42.33	12.76	141.22	198.10		
	Tenis	174.22	39.61	11.94	147.61	200.83		
IRDrel (%BW)	Baseball	1.80	0.48	0.14	1.48	2.12	1.25	0.31
	MMA	2.14	0.43	0.17	1.69	2.58		
	Swimming	2.09	0.51	0.15	1.74	2.43		
	Tenis	2.12	0.37	0.11	1.87	2.37		
ERD (N)	Baseball	161.48	36.90	11.13	136.69	186.27	0.51	0.68
	MMA	171.04	38.69	15.79	130.44	211.64		
	Swimming	163.11	22.02	6.64	148.32	177.90		
	Tenis	152.93	23.59	7.11	137.08	168.78		
ERDrel (%BW)	Baseball	1.89	0.28	0.09	1.70	2.08	1.10	0.36
	MMA	2.12	0.40	0.16	1.70	2.54		
	Swimming	2.01	0.25	0.07	1.84	2.17		
	Tenis	1.88	0.30	0.09	1.67	2.08		
IRD:ERD	Baseball	0.96	0.24	0.07	0.79	1.12	1.40	0.26
	MMA	1.01	0.10	0.04	0.91	1.11		
	Swimming	1.05	0.22	0.07	0.90	1.19		
	Tenis	1.15	0.25	0.08	0.98	1.32		

Note: IRD, shoulder internal rotational strength for dominant limb; ERD, shoulder external rotational strength for dominant limb; IRD_rel_, shoulder internal rotational strength to body weight for dominant side; ERD_rel_, shoulder rotational strength normalized to body weight for dominant side; IRD: ERD, shoulder rotational strength ratio between external and internal rotation.

### 3.3 Relationship between isometric trunk and isometric shoulder rotational strength

Isometric trunk rotational strength significantly correlated between dominant and non-dominant side in each group of athletes (baseball: *r* = 0.94, *R* ^2^ = 0.88, MMA: *r* = 0.87, *R* ^2^ = 0.76, swimming: *r* = 0.88, *R* ^2^ = 0.77, tennis: *r* = 0.74, *R* ^2^ = 0.55). We found significant correlation between internal and external SRS in MMA athletes (*r* = 0.91, *R* ^2^ = 0.83) (Table 4).

**TABLE 4:** Correlation between trunk and shoulder parameters in MMA (*n* = 6).

MMA	TRD	TRN	TRdiff	TRDrel	TRNrel	IRD	IRDrel	ERD	ERDrel	IRD_ERD
TRD	1.00									
TRN	0.87*	1.00								
TRdiff	0.34	−0.16	1.00							
TRDrel	0.94**	0.95**	0.04	1.00						
TRNrel	0.59	0.72	−0.34	0.76	1.00					
IRD	0.89*	0.84*	0.19	0.81	0.52	1.00				
IRDrel	0.80	0.81*	−0.02	0.79	0.75	0.93**	1.00			
ERD	0.88*	0.92**	0.08	0.87*	0.64	0.92**	0.87*	1.00		
ERDrel	0.75	0.88*	−0.17	0.83*	0.87*	0.77	0.87*	0.91*	1.00	
IRD:ERD	0.22	0.03	0.21	0.07	−0.06	0.44	0.42	0.06	−0.08	1.00

Note: TRD, trunk rotational strength for dominant side; TRN, trunk rotational strength for non-dominant side; TRDrel, trunk rotational strength to body weight for dominant side; TRNrel, trunk rotational strength normalized to body weight for non-dominant side; TRdiff, trunk rotational strength asymmetry between dominant and non-dominant side; IRD, shoulder internal rotational strength for dominant limb; ERD, shoulder external rotational strength for dominant limb; IRDrel, shoulder internal rotational strength to body weight for dominant side; ERDrel, shoulder rotational strength normalized to body weight for dominant side, IRD: ERD, shoulder rotational strength ratio between external and internal rotation. **p < .01, *p < .05.

Interestingly in Table 5, significant relationship has been detected between TRdiff and isometric IRD in tennis players (*r* = 0.63, *R* ^2^ = 0.40), while in another athletes this association was insignificant (*p* > 0.05).

**TABLE 5:** Correlation between trunk and shoulder parameters in tennis (*n* = 11).

Tennis	TRD	TRN	TRdiff	TRDrel	TRNrel	IRD	IRDrel	ERD	ERDrel	IRD_ERD
TRD	1.00									
TRN	0.74**	1.00								
TRdiff	−0.30	0.09	1.00							
TRDrel	0.88**	0.54	−0.46	1.00						
TRNrel	0.81**	0.88**	−0.18	0.83**	1.00					
IRD	−0.02	0.49	0.63*	−0.26	0.18	1.00				
IRDrel	0.08	0.51	0.45	0.02	0.40	0.89**	1.00			
ERD	−0.01	0.38	0.09	−0.09	0.26	0.35	0.37	1.00		
ERDrel	0.02	0.22	−0.23	0.19	0.37	−0.02	0.20	0.86**	1.00	
IRD:ERD	0.00	0.23	0.53	−0.20	0.01	0.77**	0.67*	−0.32	−0.59	1.00

Note: TRD, trunk rotational strength for dominant side; TRN, trunk rotational strength for non-dominant side; TRDrel, trunk rotational strength to body weight for dominant side; TRNrel, trunk rotational strength normalized to body weight for non-dominant side; TRdiff, trunk rotational strength asymmetry between dominant and non-dominant side; IRD, shoulder internal rotational strength for dominant limb; ERD, shoulder external rotational strength for dominant limb; IRDrel, shoulder internal rotational strength to body weight for dominant side; ERDrel, shoulder rotational strength normalized to body weight for dominant side; IRD: ERD, shoulder rotational strength ratio between external and internal rotation. **p < .01, *p < .05.

Results revealed, that the stronger isometric TRS, the stronger isometric SRS in swimmers (Table 6), MMA and baseball pitchers. But these relationships were not confirmed in tennis athletes. This relationship was insignificant for rest groups (baseball: *r* = 0.52, *R* ^2^ = 0.27, swimming: *r* = 0.51, *R* ^2^ = 0.26, tennis: *r* = 0.35, *R* ^2^ = 0.12).

**TABLE 6:** Correlation between trunk and shoulder parameters in swimmers (*n* = 11).

Swimming	TRD	TRN	TRdiff	TRDrel	TRNrel	IRD	IRDrel	ERD	ERDrel	IRD_ERD
TRD	1.00									
TRN	0.88**	1.00								
TRdiff	0.05	−0.10	1.00							
TRDrel	0.92**	0.77**	−0.07	1.00						
TRNrel	0.79**	0.88**	−0.26	0.84**	1.00					
IRD	0.68*	0.74**	−0.21	0.64*	0.66*	1.00				
IRDrel	0.63*	0.66*	−0.26	0.65*	0.67*	0.98**	1.00			
ERD	0.70*	0.82**	−0.24	0.69*	0.71*	0.51	0.46	1.00		
ERDrel	0.60	0.69*	−0.36	0.71*	0.74**	0.44	0.45	0.94**	1.00	
IRD:ERD	0.31	0.37	−0.07	0.22	0.26	0.83**	0.81**	0.00	−0.09	1.00

Note: TRD, trunk rotational strength for dominant side; TRN, trunk rotational strength for non-dominant side; TRDrel, trunk rotational strength to body weight for dominant side; TRNrel, trunk rotational strength normalized to body weight for non-dominant side; TRdiff, trunk rotational strength asymmetry between dominant and non-dominant side; IRD, shoulder internal rotational strength for dominant limb; ERD, shoulder external rotational strength for dominant limb; IRDrel, shoulder internal rotational strength to body weight for dominant side; ERDrel, shoulder rotational strength normalized to body weight for dominant side; IRD: ERD, shoulder rotational strength ratio between external and internal rotation. **p < .01, *p < .05.

Conversely, TRdiff were significantly associated with IR:ER ratio just in baseball players, as seen in Table 7 (*r* = 0.67, *R* ^2^ = 0.45).

**TABLE 7:** Correlation between trunk and shoulder parameters in baseball (*n* = 11).

Baseball	TRD	TRN	TRdiff	TRDrel	TRNrel	IRD	IRDrel	ERD	ERDrel	IRD_ERD
TRD	1.00									
TRN	0.94**	1.00								
TRdiff	−0.06	0.18	1.00							
TRDrel	0.77**	0.64*	−0.15	1.00						
TRNrel	0.88**	0.84*	−0.03	0.86**	1.00					
IRD	0.59	0.66*	0.52	0.48	0.40	1.00				
IRDrel	0.38	0.40	0.45	0.53	0.28	0.91**	1.00			
ERD	0.81**	0.80**	−0.18	0.49	0.57	0.52	0.29	1.00		
ERDrel	0.61*	0.54	−0.41	0.58	0.47	0.36	0.31	0.88**	1.00	
IRD:ERD	0.13	0.17	0.67*	0.25	0.06	0.78**	0.86**	−0.12	−0.20	1.00

Note: TRD, trunk rotational strength for dominant side; TRN, trunk rotational strength for non-dominant side; TRDrel, trunk rotational strength to body weight for dominant side; TRNrel, trunk rotational strength normalized to body weight for non-dominant side; TRdiff, trunk rotational strength asymmetry between dominant and non-dominant side; IRD, shoulder internal rotational strength for dominant limb; ERD, shoulder external rotational strength for dominant limb; IRDrel, shoulder internal rotational strength to body weight for dominant side; ERDrel, shoulder rotational strength normalized to body weight for dominant side; IRD: ERD, shoulder rotational strength ratio between external and internal rotation. **p < .01, *p < .05.

## 4 Discussion

Positive correlations were observed between TRS parameters and SRS performance across all sports, suggesting that strong and coordinated trunk rotation is a common denominator among successful athletes in MMA, tennis, swimming and baseball pitchers. However, the strength of this correlation varied between disciplines. This highlights the complex interaction between these two factors of rotational strength and the need for sport-specific training approaches.

When analysing each sport discipline, tennis players exhibited a strong correlation between TRS asymmetry and internal SRS. This finding aligns with the long-term unilateral demands of the sport, where a forceful rotation of the torso is imperative for delivering powerful serves and groundstrokes in overhead arm extension. Thus, tennis players are exposed to one-sided maladaptation (Patel et al., 2020), leading to higher interconnected shoulder and trunk rotational muscular imbalance, and consequently to potential shoulder or lower back injury (Dines et al., 2015; Zemkova, 2018). The results found in this study corroborated this, and therefore, there is a necessity to integrate training programs that address imbalances of the trunk and shoulder strength while continuing to maximize performance (Zemkova et al., 2018). The results found a very balanced trunk and shoulder strength for MMA fighters, particularly in shoulder strength, with stronger TRS associated with stronger SRS. Strength is a key factor for MMA fighters, as Folhes et al. (2022) found an association between competition level and strength performance. However, different fighting styles can have different demands, and MMA is multifaceted, where athletes engage in a combination of punches and grappling techniques from judo, jujitsu, and wrestling (Pujszo and Adam, 2016). Additionally, certain combat styles require different movement patterns and fitness demands (Iwai et al., 2008; Ambroży et al., 2021). The varying demands of these techniques may contribute to a more diversified pattern of strength development in the trunk and shoulders. Thus, these athletes had the least imbalance between shoulder IRD and ERD. Furthermore, they reached the highest relationships between TRS in non-dominant side and external SRS among all other sports. This may indicate the importance of defensive movements to prevent opponents’ dangerous actions, when MMA fighters not only exert upper limb motion in forward directions like punching, where internal shoulder rotation of dominant arm finds its support in dominant side trunk rotation (serape effect), but during torso rotation to non-dominant side they perform upper limb blocking action where external rotation plays a key role. These results showed for MMA that symmetry can be a key aspect in order to be able to be offensive with and defensive against different fighting styles that require different demands. Swimmers had shown the most significant relationships between shoulder and trunk performance overall. This complex synergy is important to develop the “swimming shoulder kinetic chain” for performance purposes (Bradley et al., 2019). Swimmers did not have a significant TRS asymmetry which indicates swimmers are well balanced in TRS. This makes sense as swimming is a symmetrical sport without a dominant side. The power generated by the shoulder during swimming can be seen by swimmers having the largest IRD shoulder strength of the sports disciplines studied. This large shoulder strength could have issues if not balanced by the TRS, as a lower contribution of trunk stabilizing muscles during swimming can lead to shoulder pain and injury (Heinlein and Cosgarea, 2010; Matsuura et al., 2022). Coaches can optimise performance of their swimmers and reduce injury risk by analysing the shoulder-trunk relationships. Baseball pitchers showed the strongest associations between TRS asymmetry and internal to external SRS ratio, while also reached high relationship between both sides of TRS and external SRS. Pitching in baseball requires greater shoulder external rotation, which helps increase pitch speed without increasing overall joint torques (Albiero et al., 2021). Furthermore, the art of pitching requires a kinematic chain that involves the entire body, in particular upper trunk rotation (Diffendaffer et al., 2022). The results and tests presented here could additionally aid professionals in baseball to highlight areas of strength improvement of the shoulder and trunk, which can be indicated in instances of poor performance or insufficient movements patterns (Diffendaffer et al., 2022). Furthermore, it could assist in injury prevention strategies, where external rotation performance is a lowering injury risk factor (Stodden et al., 2001; Hurd and Kaufman, 2012; Diffendaffer et al., 2022; Huang et al., 2022). One methodological point from this study was the expression of trunk strength relative to body weight. It may be crucial for precise assessment and fair benchmarking across athletes due to unique biomechanics and body composition. When choosing between strength tests, two main forms exist: isokinetic and isometric. They both have positive and negatives for their choice. Elite male tennis players demonstrated approximately 63% peak torque to body weight at an angular velocity of 60 s^−1^ during seated dynamic performance, with no significant difference between sides (Ellenbecker and Roetert, 2004). We found lower mean result (approximately 30%BW) within isometric “pallof” hold during standing rotational power, which may be more specific for athletic performance (Zemkova, 2018). Static tests may underestimate trunk strength and fail to capture spinal health accurately during dynamic motion (Bayramoglu et al., 2001; Zemkova, 2018). Nonetheless, static tests can aid in assessing spinal health by mitigating shear stresses and torsional compression, critical factors in joint injury mechanisms (McGill and Hoodless, 1990). These stresses, in combination with appropriate extensor torque, are of a size that should be considered in the mechanism of joint injury (McGill and Hoodless, 1990). It’s crucial to recognize that various factors such as equipment, muscle lengths, motion axis direction, patient position, and static vs. dynamic protocols can influence test outcomes (Bayramoglu et al., 2001). Dynamic tests likely reveal individual motor strategies more effectively, while isometric tests may assist in standardizing conditions for maximal strength assessment. The main limitation of the study is the small number of participants, particularly in the MMA group, due to the limited availability of elite athletes in this specific discipline in the Czech Republic. This poses a significant challenge for researchers analysing such a unique population. To improve future participant recruitment, a strategy to collaborate with different sports organizations and implement long-term monitoring may be helpful. However, the selection of participants in certain sports may be limited by the small number of athletes who compete at the highest national and international levels. In this study, we contacted sports federations and coaches to explore the possibility of measuring and analysing strength. On the other hand, we focused just on high level athletes from disciplines where core and upper body strength play a pivoting role. Based on athletes’ classification framework (McKay et al., 2022), all athletes were recruited at least from Tier 3 (Highly Trained/National Level athletes, ∼0.014% of the global population), but most of them were from Tier 2 (Elite/International Level, ∼0.0025% of the population). In order to improve the study, we recommend incorporating dynamic tests in a larger range of motion for athletes, in addition to the use of isometric tests for assessing rotational motion in the trunk and shoulder. This will provide a more comprehensive assessment of the athletes’ abilities. It is important to note that the use of isometric tests during rotational motion may be limiting from certain perspectives. Future research should use the same load cell and sampling rate for different strength tests to avoid deviations in sensitivity. Moreover, future research should examine the potential implications of imbalances or deficiencies in these strength relationships according to lower back pain and injuries; in various performance levels; different genders and maturation status across another sporting disciplines. We acknowledge the limited number of athletes in our study. As this is a pilot study focusing on a specific population, it is important for future research to continue examining and expanding the sample size. This research aims to highlight the significance and potential differences in relationships between various sports, and a larger sample size is highly recommended for future studies.

## 5 Conclusion

We examined the differences in rotational strength of the trunk and shoulders, as well as the interactions between these variables, in adult elite athletes from various sports. While no differences in peak force variables were found, unique relationships between trunk and shoulder rotational performance were discovered in MMA, tennis, swimming, and baseball. The results suggest a long-term sport-specific adaptation of the trunk-shoulder interaction in sports that require upper limb power movements. It seems, that the relationship between the various parameters of trunk and shoulder was influenced by the movement stereotype of each sport. Therefore, recognition of sport-specific interactions is critical to the development of effective training programs that enhance performance and potentially reduce injury risk in different sports. Researchers and practitioners should focus on longitudinally monitoring fluctuations in TRS and SRS relationships throughout each sport season and examining potential associations with injury incidence.

## Data Availability

The original contributions presented in the study are included in the article/Supplementary material, further inquiries can be directed to the corresponding author.
